# Plasmonic Nanoparticle-based Hybrid Photosensitizers with Broadened Excitation Profile for Photodynamic Therapy of Cancer Cells

**DOI:** 10.1038/srep34981

**Published:** 2016-10-11

**Authors:** Peng Wang, Hong Tang, Peng Zhang

**Affiliations:** 1 Department of Chemistry, University of Cincinnati, Cincinnati, OH 45221, USA

## Abstract

Photodynamic therapy combining nanotechnology has shown great potential with improved therapeutic efficacy and fewer side effects. Ideal photosensitizers for cancer treatment should both have good singlet oxygen production capability and be excitable by light illuminations with deep tissue penetration. Here we report a type of hybrid photosensitizers consisting of plasmonic silver nanoparticles and photosensitizing molecules, where strong resonance coupling between the two leads to a broadened excitation profile and exceptionally high singlet oxygen production under both visible light and infrared light excitations. Our results indicate that the hybrid photosensitizers display low cytotoxicity without light illumination yet highly enhanced photodynamic inhibition efficacy against Hela cells under a broad spectrum of light illuminations including the near-infrared light, which has great implication in photodynamic therapy of deep-tissue cancers.

Cancer, the most frequent form of malignant diseases worldwide, is currently the leading cause of death in the world^1^. Over the decades, battles against cancer have led to the discovery of many new strategies to treat cancers. Among them, photodynamic therapy (PDT) represents a therapeutic modality currently approved by FDA for clinical treatment of several types of cancers and non-oncological diseases^2^,^3^. Photodynamic therapy is based on the induction of cell death by reactive oxygen species (ROS) produced by light illumination on the so-called photosensitizer in the presence of oxygen. Photosensitizer is first delivered to the target tissues to be treated, subsequently photoactivated by illumination of light at certain wavelengths. Energy transfer between the activated photosensitizer and oxygen molecules generates ROS, such as singlet oxygen (^1^O_2_), which starts a cascade of biochemical events to induce cytotoxicity of neoplastic cells and the regression of tumor^4^,^5^,^6^,^7^.

At the present, PDT efficacy is limited by factors such as the photochemical property of photosensitizer, delivery of photosensitizer to the target, and the penetration depth of light used to activate the photosensitizer^8^. The combinations of PDT with other therapeutic modalities have become an attractive approach in cancer therapy^9^,^10^. Multifunctional nanomaterials with novel and intrinsic physical properties have emerged as promising candidates for cancer diagnosis and therapy^11^,^12^,^13^,^14^,^15^. In recent years, a series of nanoplatform-based photosensitizer have been developed with promising features such as targeting delivery, controlled release, and deep tissue penetration^16^,^17^,^18^,^19^. To the other end, it has been known that, when some light-absorbing molecules are brought close to certain metal nanoparticles, the plasmon-molecular resonance coupling between the metal nanoparticle and the small molecules would lead to strong light absorption by the hybrid nanostructures, which has been exploited as a new direction in the development of photonic devices and optically responsive or active nanomaterials^20^,^21^,^22^. Metal-molecule hybrid nanostructures have also been proposed theoretically to enhance photochemical production in the photosynthetic systems^23^. Still, to our best knowledge, few studies focus on the exploitation of these properties for photodynamic therapy against cancers^24^,^25^,^26^.

Here we report a type of hybrid photosensitizers consisting of plasmonic silver nanoparticles coated with mesoporous silica (mSiO_2_), and photosensitizing molecules, using hematoporphyrin IX (HPIX) as a model, where strong resonance coupling between the two leads to exceptionally high singlet oxygen production under broad spectral excitations. At the dosage level where these hybrid photosensitizers display little cytotoxicity without light illumination, they can effectively inhibit Hela cells under both visible and red/near-infrared illuminations. Several factors affecting the inhibition efficiency of cancer cells have been considered and investigated for potential cancer treatment applications.

## Results and Discussion

### Synthesis and characterizations of Ag@mSiO_2_@HPIX hybrids

The synthesis of Ag@mSiO_2_@HPIX hybrids is similar to our previous reports^24^,^25^. The mesoporous silica surrounding the silver nanoparticles can host a variety of photosensitizers, including those not soluble in aqueous media. A typical transmission electron microscopy (TEM) image of Ag@mSiO_2_ nanoparticles (Figure S1) shows that they are fairly monodispersed with a diameter of 55 ± 5 nm (n = 60). The thickness of the mesoporous SiO_2_ shell is ~16 nm and the diameter of silver nanoparticles core is ~25 nm. The loading efficiency of HPIX in the hybrids was determined experimentally as shown in Figure S2, and calculated to be ~1.15 μmol of HPIX per 100 mg of Ag@mSiO_2_.

### Plasmon-photosensitizer resonance coupling effect

**Figure 1:**
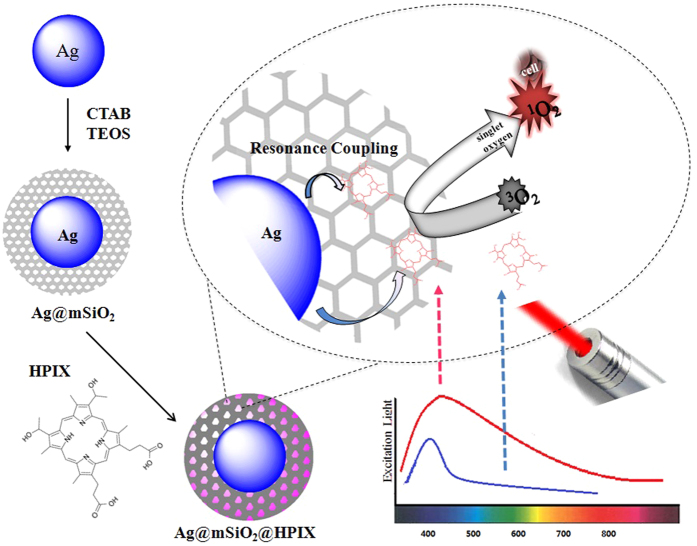
Schematic of the synthesis of Ag@mSiO_2_@HPIX hybrid and its photodynamic action against cancer cells.

The schematic of the photodynamic action of Ag@mSiO_2_@HPIX hybrids against cancer cells is illustrated in Fig. 1. HPIX molecules are brought close to the silver nanoparticle core by the mesoporous silica layer. In a previous study^24^, we had shown that HPIX displayed the best singlet oxygen production enhancement in this type of nanostructures among a number of tested photosensitizers. Therefore, we chose to use HPIX in this study. Consistent with the previous study, the Ag@mSiO_2_@HPIX hybrids display synergistic effect in singlet oxygen production because of the plasmon-photosensitizer resonance coupling between the silver nanoparticle and HPIX. Experimentally, this is demonstrated by measuring their singlet oxygen production under both visible light (400 nm) and near-infrared light (850 nm) excitations for Ag@mSiO_2_@HPIX, free HPIX, and Ag@mSiO_2_. The concentrations of HPIX and Ag in the latter two were adjusted to be the same as their counterparts in the hybrids.

**Figure 2:**
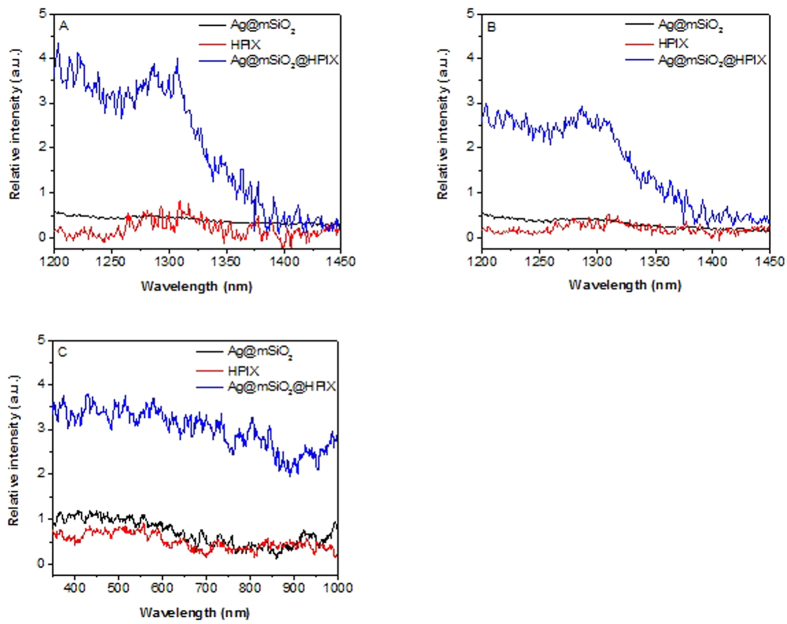
Singlet oxygen phosphorescence spectra of Ag@mSiO_2_@HPIX, free HPIX and Ag@mSiO_2_ under 400 nm (**A**) and 850 nm (**B**) excitations. (**C**) Singlet oxygen excitation spectra of Ag@mSiO_2_@HPIX, free HPIX, and Ag@mSiO_2_. Solutions of Ag@mSiO_2_@HPIX and free HPIX have the same concentration of HPIX, while solutions of Ag@mSiO_2_@HPIX and Ag@mSiO_2_ have the same concentration of Ag.

Singlet oxygen was monitored spectroscopically at its emission peak of ~1280 nm. Enhancement factor (EF) of singlet oxygen production is defined as EF = (E_h_ − E_p_ − E_Ag_)/E_p_, where E_h_, E_p_, and E_Ag_ are the singlet oxygen emission intensity of Ag@mSiO_2_@HPIX, free HPIX, and Ag@mSiO_2_, respectively. As shown in Fig. 2A, under 400 nm excitation, Ag@mSiO_2_@HPIX hybrids have a much higher singlet oxygen production than that of free HPIX or Ag@mSiO_2_ separately, with an enhancement factor of 3.1. More remarkably, significant singlet oxygen production can be observed from Ag@mSiO_2_@HPIX hybrids under near-infrared excitation at 850 nm (Fig. 2B), with the singlet oxygen enhancement factor increased to 4.2. Note that there is almost no singlet oxygen produced by the free HPIX under 850 nm excitation. However, singlet oxygen production from the Ag@mSiO_2_@HPIX hybrids only reduces by ~30% under 850 nm excitation as compared to that under 400 nm excitation. The excitation spectra of singlet oxygen production shown in Fig. 2C directly illustrate the broadened excitation profile (350–900 nm) of the Ag@mSiO_2_@HPIX hybrids, similar to our previous study involving a different photosensitizer^25^. These results strongly support that the design of the plasmon-photosensitizer hybrids in general can serve as an effective platform to broaden the excitation profile of free photosensitizers, and to allow their use under a wide range of light excitations including the near-infrared light.

### Cytotoxicity of Ag@mSiO_2_@HPIX on Hela cells without light illumination

**Figure 3:**
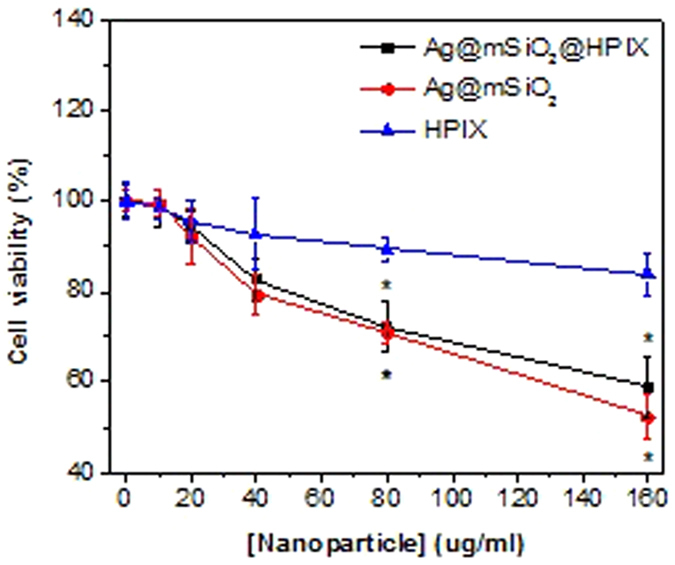
Viability of Hela cells treated with different concentrations of Ag@mSiO_2_@HPIX, free HPIX, and Ag@mSiO_2_, respectively, without light illumination for 24 h. Significance (*) was based on p < 0.05 (N = 6) against the control group.

We first investigate the cytotoxicity of the Ag@mSiO_2_@HPIX hybrids on Hela cells without light illumination. The viabilities of Hela cells treated with different concentrations of Ag@mSiO_2_@HPIX, Ag@mSiO_2_, or free HPIX, respectively, for 24 h were determined by the MTT assay. As shown in Fig. 3, Ag@mSiO_2_@HPIX displayed a significant inhibition effect on the proliferation of Hela cells at 80–160 μg/mL. At concentrations ≤20 μg/mL, Ag@mSiO_2_@HPIX showed little effect on the Hela cell viability (cell viability > 90%). Cytotoxicity of free HPIX and Ag@mSiO_2_, individually, was also determined, where their concentrations were adjusted to be the same as their counterparts in the hybrids. Note that free HPIX did not display significant inhibition effect even at the highest tested concentration (cell viability > 80%), while Hela cells were sensitive to Ag@mSiO_2_ at concentrations of 80–160 μg/mL. Accordingly, we chose 20 μg/mL of Ag@mSiO_2_@HPIX for the subsequent PDT assays, where the cytotoxicity without light illumination was negligible.

### PDT effect of Ag@mSiO_2_@HPIX hybrids on cancer cells

**Figure 4:**
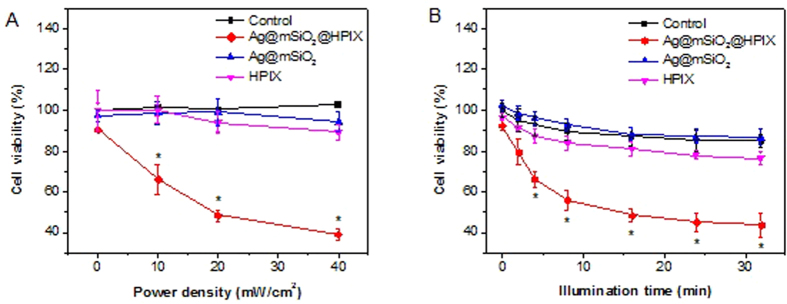
Viability of Hela cells incubated with 20 μg/mL of Ag@mSiO_2_@HPIX, free HPIX, and Ag@mSiO_2_. (**A**) Illuminated under different density of white light for 8 min. (**B**) Illuminated under 20 mW/cm^2^ of white light for different durations. Significance (*) was based on p < 0.05 (N = 6) against the control group.

We investigated the power dependence of the PDT effect of Ag@mSiO_2_@HPIX on Hela cells. After incubation with 20 μg/mL of Ag@mSiO_2_@HPIX for 24 h, Hela cells were illuminated by white light of different power densities for a total of 8 min, pausing for 30 s after 4 min to minimize any photothermal effect. As shown in Fig. 4A, the percentage of surviving cells decreased as the power density of illumination increased, and the cell viability decrease was significant even at the lowest tested power density. In contrast, viability of Hela cells treated with Ag@mSiO_2_ or free HPIX, respectively, under the same illumination conditions did not have significant change (Fig. 4A). These results demonstrate that Ag@mSiO_2_@HPIX hybrids can be activated by white light illumination to inhibit cancer cells.

We next investigated the dependence of illumination time for the PDT effect. Hela cells were treated with 20 μg/mL of Ag@mSiO_2_@HPIX, and illuminated by white light of 20 mW/cm^2^. We varied the illumination time from 2 to 32 min, with a 30 s-pause after every 4 min of illumination. Results in Fig. 4B showed that the longer the illumination time, the higher the cell inhibition efficiency. Compared with Hela cells treated by Ag@mSiO_2_ or free HPIX, respectively, under the same illumination conditions, Ag@mSiO_2_@HPIX displayed a significant enhancement in the inhibition efficiency of cell viability. These results are consistent with the singlet oxygen productions of Ag@mSiO_2_@HPIX, free HPIX, and Ag@mSiO_2_ as shown in Fig. 2. Furthermore, the Hela cells treated with 20 μg/mL of Ag@mSiO_2_@HPIX and illuminated by white light at 20 mW/cm^2^ were stained with Trypan Blue, a dye that only stains dead cells, and imaged microscopically. The results in Figure S3 show little cell death in the non-treated controls. Only a few dead cells were observed in the non-treated cells under 16 min white light illumination and the cells treated with Ag@mSiO_2_ under 16 min white light illumination. In contrast, increasing dead cells were observed in the samples treated with Ag@mSiO_2_@HPIX hybrids under white light illumination of increasing durations (4–16 min). In addition, Figure S4 shows that cellular uptake of both free HPIX and Ag@mSiO_2_@HPIX readily occurs with Hela cells, as indicated by the fluorescence signal of HPIX. No significant difference in fluorescence is observed in the cells incubated with Ag@mSiO_2_@HPIX or free HPIX, which suggests that the difference in the PDT effect of Ag@mSiO_2_@HPIX and of free HPIX is not caused by the difference in cellular uptake of the photosensitizers. These results confirm the enhanced PDT effect induced by the presence of Ag@mSiO_2_@HPIX hybrids under white light illumination.

**Figure 5:**
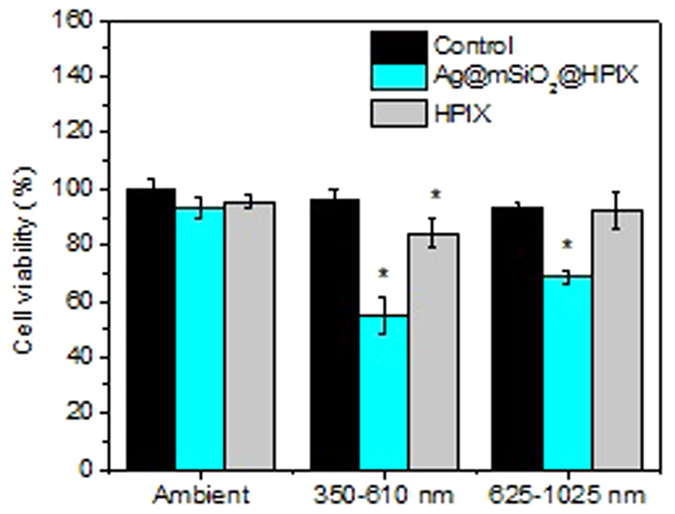
Viability of Hela cells incubated with 20 μg/mL of Ag@mSiO_2_@HPIX or free HPIX, respectively, under 20 mW/cm^2^ illumination with different wavelength range for 8 min. Significance (*) was based on p < 0.05 (N = 6) against the non-treated control.

The spectroscopic results in Fig. 2 indicate that Ag@mSiO_2_@HPIX hybrids can produce singlet oxygen under broad spectral excitations. To take advantage of this property for potential cancer treatment applications, we evaluated the hybrids’ PDT efficacy under light illuminations of two wavelength ranges (350–610 and 625–1025 nm) using different bandpass filters. In these experiments, Hela cells were treated similarly as described above, and the light density after passing through the two different filters were adjusted to be 20 mW/cm^2^. Results are shown in Fig. 5. Under the illumination of 350–610 nm light, both free HPIX and Ag@mSiO_2_@HPIX hybrids could be excited to produce singlet oxygen and inhibit Hela cells. The PDT efficacy by Ag@mSiO_2_@HPIX was much higher than that by free HPIX. In contrast, under the illumination of 625–1025 nm light, free HPIX could not be excited effectively to inhibit Hela cells, while Ag@mSiO_2_@HPIX hybrids maintained significant PDT efficacy against Hela cells. These results not only confirm the previous spectroscopic observations of the Ag@mSiO_2_@HPIX hybrids, but also illustrate their great implication to cancer treatment applications, where it is desirable to use red or near-infrared light as the illumination source, which has deeper tissue penetration.

### Evaluation of simulated PDT efficacy of Ag@mSiO_2_@HPIX against deep-tissue cancer

**Figure 6:**
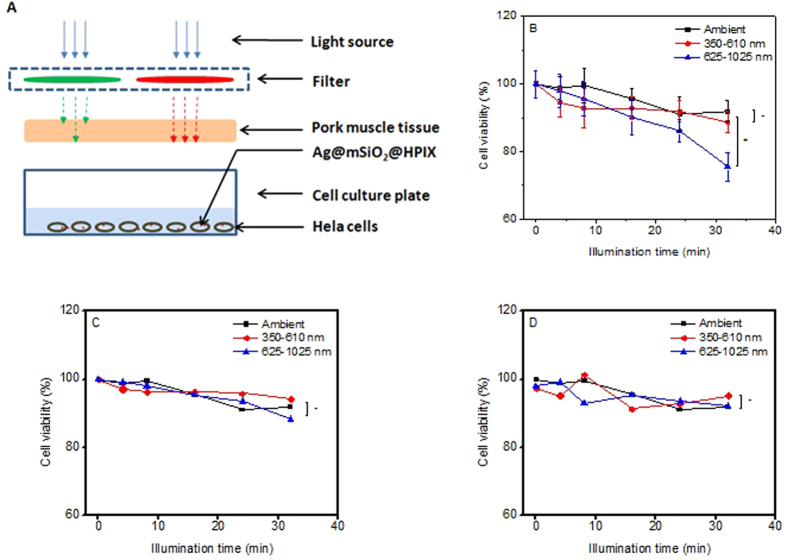
(**A**) Schematic of the setup of simulated deep-tissue condition. Viability of Hela cells treated by 20 μg/mL of Ag@mSiO_2_@HPIX (**B**), free HPIX (**C**), and Ag@mSiO_2_ (**D**) under illumination of 20 mW/cm^2^ for up to 32 min with a 1-cm thick pork muscle tissue between the light output and the cells. Significance (*) was based on p < 0.05 (n = 4) and non-significance (-) on p > 0.05 (n = 4) for pairs.

To further assess the potential of applying Ag@mSiO_2_@HPIX in the PDT treatment of deep-tissue cancers, we carried out experiments to evaluate how the presence of tissues would affect the PDT efficacy of the hybrids under different excitations. In these experiments, we followed a setup similar to what has been reported by others in the literature^16^. A piece of 1-cm thick pork muscle tissue was placed above the wells containing Hela cells incubated with 20 μg/mL of Ag@mSiO_2_@HPIX, as illustrated in Fig. 6A. PDT assays were carried out either without light illumination or under 20 mW/cm^2^ illumination of different wavelengths for up to 32 min. Cell viability was then determined similar to previously described, with results shown in Fig. 6B. We notice that there is no statistically significant difference of cell viability between the samples without light illumination and the samples under illumination of wavelength range from 350–610 nm. Under the illumination of wavelength range from 625–1025 nm light, the PDT effect was statistically significant for the sample with 32 min of illumination.

For comparison, viability of Hela cells treated with corresponding concentrations of Ag@mSiO_2_ and free HPIX under the same illumination condition were shown in Fig. 6C,D. There is no statistically significant difference of cell viability between the samples without light illumination and with light illumination within the treatment duration (up to 32 min), regardless of the wavelength range. These results demonstrate that the Ag@mSiO_2_@HPIX hybrids can function as photosensitizers under red/near-infrared excitations despite a 1-cm thick pork muscle tissue placed between the light source and the samples, which represents a major improvement in the design of photosensitizers. The capability of exciting these hybrid photosensitizers in the near-infrared region brings about enormous potential to the molecule-type photosensitizers only excitable by visible light, such as HPIX, for PDT applications.

In summary, we have demonstrated that hybrid nanostructures consisting of plasmonic silver nanoparticles coated by HPIX-loaded mesoporous silica (Ag@mSiO_2_@HPIX) can effectively serve as photosensitizers in photodynamic therapy against cancer cells. These hybrid photosensitizers exhibit significant enhancements in both singlet oxygen production and PDT efficacy against cancer cells. The excitation profile of the hybrid photosensitizers is broadened by the presence of the silver nanoparticles, allowing them to display effective photodynamic action under red/near-infrared light excitations for deep-tissue cancer treatments. The combination of low cytotoxicity without light illumination and high PDT efficacy of the hybrid photosensitizers has great implication to their potential for clinical applications.

## Methods

### Chemicals and materials

Hematoporphyrin IX dihydrochloride (HPIX) was purchased from Frontier Scientific. Tetraethyl orthosilicate (TEOS), 3-(4,5-Dimethyl-2-thiazolyl)-2,5-diphenyl-2H-tetrazolium bromide (MTT), Trypan Blue solution, silver nitrate, and absolute anhydrous ethanol were from Sigma Aldrich. Ammonium nitrate, nitric acid (68%), formaldehyde solution (37%), cetyltrimethylammonium bromide (CTAB), sodium hydroxide, sodium cyanide, Penicillin-Streptomycin (10,000 U/mL), DAPI (4′,6-diamidino-2-phenylindole), and PBS buffer were from Thermo Fisher. All chemicals were used as received. Human cervical cancer cells Hela (ATCC CCL-2) were obtained from American Type Culture Collection (ATCC). Dulbecco’s Modified Eagle’s Medium (DMEM) was purchased from Sigma, and fetal calf serum (FBS) from Hyclone.

### Synthesis of silver-mesoporous silica core-shell nanoparticles

The synthesis of silver-mesoporous silica core-shell nanoparticles (Ag@mSiO_2_) is similar to what was reported previously^24^,^25^. Typically, 20 mg of CTAB was dissolved in a solution containing 0.24 mL of 0.5 M NaOH and 9.8 mL of water. After stirring the mixture at 80 ^o^C for 10 min, 0.24 mL of 0.1 M silver nitrate solution and 60 μL of 1.0 M formaldehyde solution were added quickly. Next, 70 μL TEOS was added at a rate of 3 mL/h using a syringe pump. The reaction was allowed to proceed at 80 ^o^C under stirring for 2 h. Thereafter, the products were centrifuged and washed by ethanol. Finally, the CTAB component in the nanoparticles was removed by extraction in ethanol solution containing ammonium nitrate (6 g/L) at 50 ^o^C for 30 min.

### Synthesis of Ag@mSiO_2_@HPIX hybrids

Freshly synthesized Ag@mSiO_2_ nanoparticles were dispersed in 10 mL DI water under stirring. Then, 1 mL of 1 mM HPIX solution in ethanol was added and stirred at room temperature overnight. The products were centrifuged and the supernatant removed. Finally, the synthesized Ag@mSiO_2_@HPIX hybrids were dispersed into 10 mL DI water under sonication, and used as stock solution.

### Fluorescence and phosphorescence measurements

All fluorescence and phosphorescence spectra were collected on a QM-40 spectrofluorometer (PTI) equipped with an R928 PMT and a high performance InGaAs photodiode as detectors. Detection of singlet oxygen production was done by monitoring its phosphorescence emission at ~1280 nm. The light source was a Xenon arc lamp. An additional long-pass filter (850 nm cut-off) was placed after the sample holder to remove any possible higher-order artifact signals. The fluorescence excitation and emission peaks for HPIX were 397 nm and 620 nm, respectively.

### TEM measurement

TEM measurements were done on a Biotwin 12 transmission electron microscope. A drop of the sample aqueous solution was applied onto a carbon-coated copper grid (300 mesh, EMS) and left dried for measurement.

### Cell culture

Hela cells (ATCC CCL-2) were maintained in DMEM supplemented with 10% FBS, 100 unit/mL penicillin and 0.1 mg/mL streptomycin at a 37 °C incubator with a 5% CO_2_ atmosphere.

### Cytotoxicity test of Ag@mSiO_2_@HPIX hybrids

Cytotoxicity of Ag@mSiO_2_@HPIX hybrids to Hela cells were evaluated using a standard methyl thiazolyl tetrazolium (MTT) assay. Briefly, Hela cells were seeded in 96-well plates containing 100 μL medium at a density of ~1.0 × 10^4^ cells per well. After being incubated overnight, the cells were treated for 24 h with various concentrations (0, 10, 20, 40, 80, and 160 μg/mL) of Ag@mSiO_2_@HPIX in DMEM supplemented with 10% FBS at 37 °C under 5% CO_2_. Subsequently, MTT in PBS solution (5 mg/mL) was added to the culture medium to reach a final concentration of 0.5 mg/mL. After the cells were incubated at 37 °C for 4 h, the supernatants were removed and the formazan dye was dissolved in 100 μL DMSO. Absorbance was measured on a microplate reader at 490 nm with a reference wavelength at 650 nm. Absorbance values of all samples were offset by the absorbance of the medium control. Absorbance of the non-treated controls was set as 100% to calculate cell viability (%).

### Photodynamic effect of Ag@mSiO_2_@HPIX hybrids

To evaluate the photodynamic effect of the Ag@mSiO_2_@HPIX hybrids on cancer cells, Hela cells were seeded onto a 96-well plate at a density of ~1.0 × 10^4^ cell/well and incubated for 24 h. Then the medium was replaced by a medium solution (100 μL/well) containing 20 μg/mL of Ag@mSiO_2_@HPIX hybrid, Ag@mSiO_2_, or free HPIX, respectively. After incubation for 4 h, the cells were washed with PBS. Then 100 μL/well of PBS was added, and the cells were exposed to light illumination of different durations, respectively. A non-coherent, white light source with interchangeable fiber bundle (Model LC-122, LumaCare) was employed to illuminate the cells. Two bandpass filters were used to select the proper wavelength ranges, 350–610 nm (Edmund Optics, #84-905) and 625–1040 nm (Edmund Optics, #84-903). The irradiance at the position of the samples was maintained at 20 mW/cm^2^, as measured by a laser power meter (Model 840011, SPER Scientific). The cells were illuminated intermittently, paused for 30 s after every 4 min, to minimize the potential photothermal effect on the cells. For each experiment, non-treated controls were included and controls without illumination were run in parallel. After illumination, PBS was replaced by the culture medium (100 μL/well), and the cells were incubated for 24 h at 37 °C under 5% CO_2_ before evaluated with the aforementioned MTT assay. Absorbance of the non-treated sample without illumination was set as 100% to calculate cell viability (%) of other samples.

### Cell optical imaging

Cell imaging was carried out after the cells had been treated photodynamically. In brief, Hela cells were seeded onto a 24-well plate at a density of ~1.0 × 10^5^ cell/well and incubated for 24 h. Then the medium was replaced by a medium solution (0.5 mL/well) containing 0 μg/mL, 20 μg/mL of Ag@mSiO_2_, and 20 μg/mL of Ag@mSiO_2_@HPIX hybrid, respectively. After incubation for 4 h, the cells were washed with PBS. Then 0.5 mL/well of PBS was added, and the cells were exposed to light illumination as previously described for 10 min. Afterwards, PBS was replaced by the culture medium (100 μL/well), and the cells were incubated for 24 h at 37 °C under 5% CO_2_ before being stained with Trypan Blue and washed with PBS, and imaged on a Nikon EXFO X-Cite 120 microscope with a 40X objective.

### Fluorescence imaging of cellular uptake of photosensitizers

To study the cellular uptake of Ag@mSiO_2_@HPIX and free HPIX, Hela cells were seeded on a cover glass placed in a 6-well plate at a density of ~1.0 × 10^5^ cell/well in DMEM. After incubation for 24 h, the culture medium was replaced by the same medium containing 20 μg/mL Ag@mSiO_2_@HPIX or equivalent concentration of free HPIX. After incubation for 5 h, the cell medium was removed, and the cells were washed three times with PBS. The cells were then stained with 2 μg/mL DAPI solution before being imaged on a Nikon A1 multiphoton confocal microscope with a 40X objective.

### Statistical analysis

Data are shown as means ± standard deviation. Differences are analyzed for statistical significance by the two-sample t-test and a p-value of < 0.05 is considered significant. All experiments were run with duplicate or triplicate samples. At least two independent experiments were carried out for each experiment.

## Additional Information

**How to cite this article**: Wang, P. *et al*. Plasmonic Nanoparticle-based Hybrid Photosensitizers with Broadened Excitation Profile for Photodynamic Therapy of Cancer Cells. *Sci. Rep.* **6**, 34981; doi: 10.1038/srep34981 (2016).

## Supplementary Material

Supplementary Information
