# Supplementary material for: Plasmonic Nanoparticle-based Hybrid Photosensitizers with Broadened Excitation Profile for Photodynamic Therapy of Cancer Cells
Source: Sci Rep. 2016 Oct 11;6:34981. doi: 10.1038/srep34981 (PMC5057082; doi:10.1038/srep34981)
Supplement: Supplementary Information [file srep34981-s1.pdf]

Plasmonic Nanoparticle-based Hybrid Photosensitizers with Broadened Excitation Profile for Photodynamic Therapy of Cancer Cells

Peng Wang, Hong Tang, Peng Zhang *

Department of Chemistry, University of Cincinnati, Cincinnati, OH 45221

Supplementary information

<u>TEM image of the Ag@mSiO$_2$@HPIX hybrids</u>

A typical transmission electron microscopy (TEM) image of Ag@mSiO$_2$ nanoparticles is shown in Figure S1.

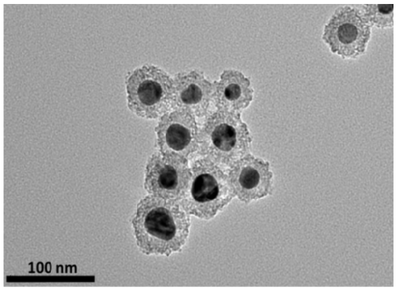

Figure S1. Representative TEM image of Ag@mSiO$_2$.

<u>Determination of loading efficiency of HPIX in Ag@mSiO$_2$</u>

First, a calibration curve of a series of concentrations of HPIX (2.5 µM, 3 µM, 3.5 µM, 4 µM, 4.5 µM, 5 µM) was obtained under excitation of 400 nm (Figure S2). The calibration curve showed a linear relationship between the fluorescence intensity of HPIX at 630 nm and the concentration of HPIX (in µM). Then, 100 µL HPIX (50 µM) was added to 1 mL of Ag@mSiO$_2$ solution and stirred at room temperature overnight before centrifugation. The supernatant was collected, fluorescence intensity of which was measured and compared to the calibration curve. The loading efficiency of HPIX was calculated to be ~1.15 µmol per 100 mg of Ag@mSiO$_2$.

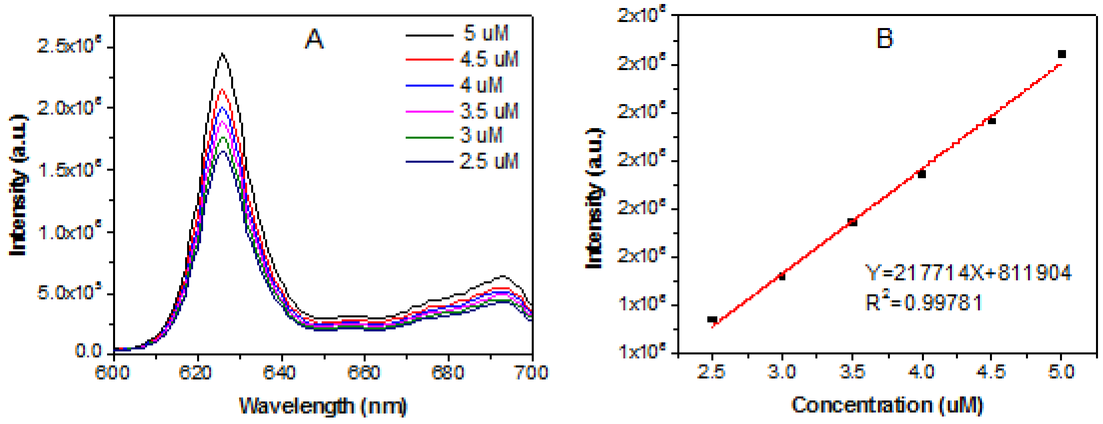

Figure S2. (A) Fluorescence emission spectra of HPIX at various concentrations. (B) The calibration curve of HPIX fluorescence intensity *vs.* its concentration.

Optical imaging of Hela cells

Cell imaging was carried out on a Nikon EXFO X-Cite 120 microscope with a 40× objective.

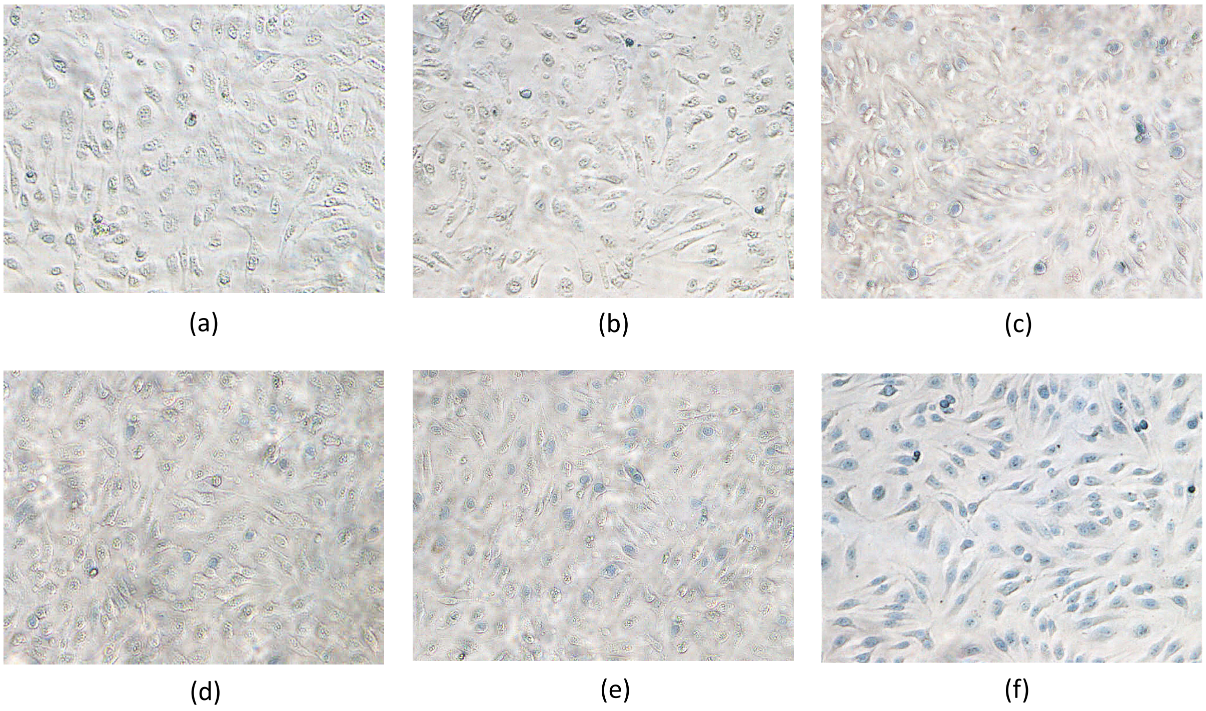

Figure S3. Optical images of Hela cells stained with Trypan Blue after different treatments (20 μg/mL Ag@mSiO$_2$@HPIX, and white light power density 20 mW/cm^2). (a) Non-treated cells with no illumination. (b) Non-treated cells under 16 min illumination. (c) Cells treated with

$Ag@mSiO_2$ under 16 min illumination. (d) Cells treated with $Ag@mSiO_2@HPIX$ under 4 min illumination. (e) Cells treated with $Ag@mSiO_2@HPIX$ under 8 min illumination. (f) Cells treated with $Ag@mSiO_2@HPIX$ under 16 min illumination.

Fluorescence imaging of Hela cells

Fluorescence imaging was carried out on a Nikon A1 multiphoton confocal microscope with a 40× objective.

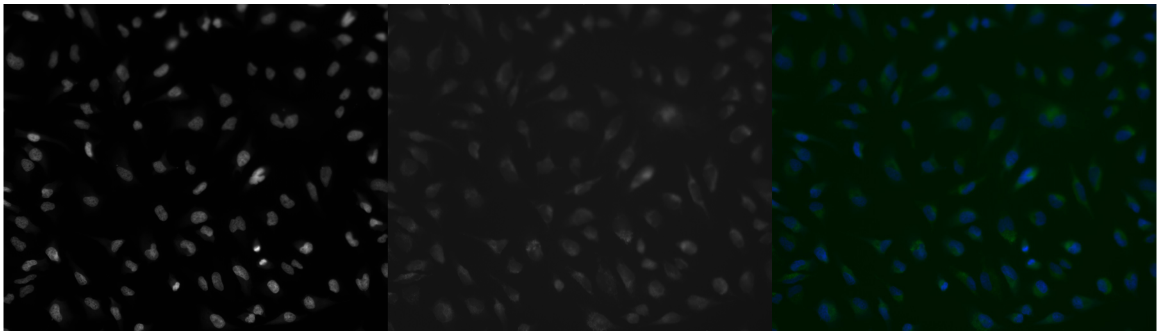

(a) (b) (c)

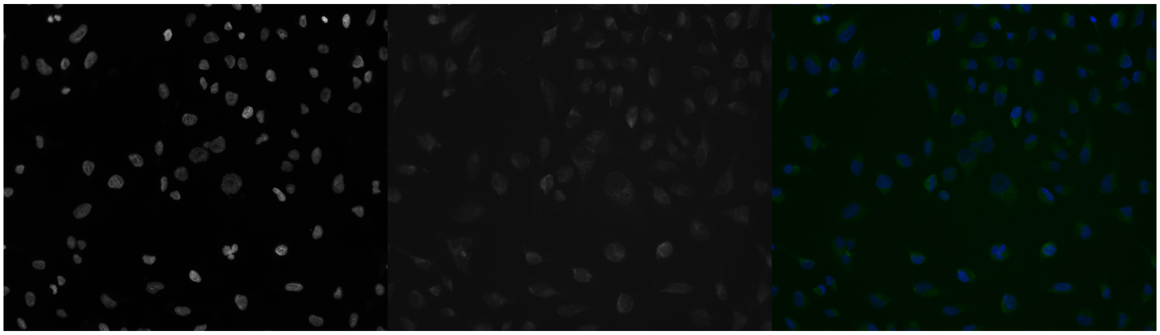

(d) (e) (f)

Figure S4. Fluorescence images of Hela cells after incubation with 20 µg/mL $Ag@mSiO_2@HPIX$ (a-c) and equivalent amount of free HPIX (d-f), respectively. The cell nuclei were stained with DAPI. (a) and (d) are DAPI fluorescence signals. (b) and (e) are HPIX fluorescence signals. (c) and (f) are the overlays of DAPI (blue) and HPIX (green).
